# Genome-Wide Profiling of Human Papillomavirus DNA Integration into Human Genome and Its Influence on PD-L1 Expression in Chinese Uygur Cervical Cancer Women

**DOI:** 10.1155/2020/6284960

**Published:** 2020-01-23

**Authors:** Feng Yang-chun, Wang Sen-yu, Zhang Yuan, Huang Yan-chun

**Affiliations:** ^1^Clinical Laboratory Center, Tumor Hospital Affiliated to Xinjiang Medical University, China; ^2^Cancer Research Institute, Tumor Hospital Affiliated to Xinjiang Medical University, China

## Abstract

**Background:**

The Uygur is the fifth most populous ethnic group in China. Compared to other Chinese population, cervical cancer in them had high incidence, and HPV infection also was particular. Their HPV integration situation has never been reported. We aimed to investigate the integration situation of 20 subtypes of HPV gene into host cell genome in Chinese Uygur cervical cancer patients; meanwhile, we explored the influence of gene integration on PD-L1 expression.

**Methods:**

40 frozen Chinese Uygur cervical cancer specimens with positive HPV infection were obtained from the cancer prevention and treatment institute of Tumor Hospital Affiliated to Xinjiang Medical University. The integration situation of HPV gene into host cell genome was detected by Agilent SureSelect™ Target Enrichment Chip and Next-Generation Sequencing. The related genes were analyzed by GO functional annotation and KEGG pathway enrichment. The expression levels of PD-L1 in cancer cells were tested by immunohistochemical assay (IHC). Meanwhile, the relationship between PD-L1 levels in cancer cells and gene integration were analyzed.

**Results:**

The HPV multiple infection rate by HIVID was as high as 92.5%, much higher than 35.0% by the commercial kit (*P* < 0.05). There were 13423 integration events in 40 specimens, involving 6867 human genes. These integration events were distributed on all human chromosomes, and chromosome 19 had the excessive concentration phenomenon of integration events. There were some integration hotspots in human genome such as PPP1R37, HECW2, EMBP1, ANKRD50, SPTBN4, LINC00895, LYRM4-AS1, LINC00374, RBFOX1, CSMD1, CDH13, and KLHL4. Insertion breakpoints can be found in all gene regions of the HPV genome. The actual observation of the integration times of E1 and E6 was much higher than the expected value, while the actual observation times of E5 were much lower than the expected value. The result of GO functional analysis showed that binding molecular function and cellular process biological process were the main ways to influence the cell biological behavior of HPV gene integration. The enrichment pathway analysis of KEGG showed that pathways in cancer were the most important enrichment pathways involved in the genomic integration of HPV. The positive PD-L1 rate was 62.5%. Logistic regression analysis showed that 9p24.1 existing integration sites and the number of all gene integration were risk factors for PD-L1 expression (odds ratio 17.313 and 1.012; 95% confidence interval 1.691-177.213 and 1.001-1.023). *Conclusions and Relevance*. Most high-frequency sites of HPV integration in Chinese Uygur cervical cancer are related to cancer progression, and the gene integration hotspots may be potential HPV carcinogenic targets. The problem of multiple HPV infection in Chinese Uygur cervical cancer patients should be paid attention. L1 and E6 genes are inapposite as the target gene of commercial HPV type detection kit, because of high-frequency breakpoints in these genes. The gene integration especially the integration existing on 9p24.1 could affect the expression level of PD-L1.

## 1. Introduction

Cervical cancer was one of the most common malignant tumors in China, in which its prevalence rate was 7th in all cancers and its mortality was 8th in all female cancers [1]. HPV infection is known as the main cause of cervical cancer; most of HPV infection could be cleaned by the host immune responses, but some persistent HPV infection would lead to the cytopathy of cervical cancer cells [2–4]. The previous studies found that the HPV persistent infection consisted of two phases: in the first phase of viral replication, HPV firstly infected basal cells of the cervix, bare virus DNA was the free state in the host cell nucleus. HPV DNA independently began to replicate through using the ingredients of host cells when basal cells were in the progress of differentiation. The complete virus would be assembled by E1-E7 proteins and L1-L2 proteins. The virus DNA encapsulated in a protein coat would release from the surface mature cervical epithelial cells which create the next infection of normal cervical cells. The second phase was the transformation stage, HPV DNA integrated into the host cell chromosome. In immature epithelial cells, HPV E6 and E7 gene began to have uncontrolled expression, and the excessive expression of E6 and E7 protein would, respectively, combine with the gene products of p53 and Rh, which led to the immaturity of cervical epithelial cells; meanwhile, the viruses could not complete the whole life cycle, the L1 protein expression was missing, the infection cells created immune escape, the virus could not be cleared by the immune system, the immaturity of cervical epithelial cells led to immortalization, and lastly, it caused the lesions and canceration of cervical cells. Due to the fact that the HPV gene into the human genome was the key step in cervical cancer occurrence, so to explore the integration sites of HPV genome into the human genome and the integrated position influence on human cell genome had important clinical significance [5–7].

The mainstream methods of HPV detection included two types according to the basic principle, such as the second-generation hybrid capture method (HC-2) and fluorescence PCR method, which could be used to differentiate the subtypes of HPV infection and evaluate the total amount of viral replication in the high-risk type HPV. But these tests only focused on whether HPV gene existed in host cells, which could not detect whether HPV genome was integrated into the human genome, could not provide the location of the HPV genome into the host genome, and could not find which segment of the viral genome could integrate into the host genome. Because HPV gene integrating into the human genome was the key step that led to the host cell pathological changes, therefore it needed to analyze the HPV gene integration status, in order to assess the potential effect of HPV infection on the infected cervical cells, and thus, it met the requirements of precision medical treatment.

Though HPV genome integrating into human genome research had been reported, most of the studies only focused on certain genes of HPV16 or HPV18 type, such as the genetic integration of HPV16 E6 and E7 genes [8–12]; the integration of whole HPV genome into the host cell genome was reported in few reports. The Chinese Uygur has over a population of 10 million. The HPV infection rate and HPV subtypes in them were obviously different from the other areas in China, and they also had the highest morbidity compared with other population [13]. So it was believed that the HPV gene integration sites should have their own characteristics in cervical cancer of Chinese Uygur women.

HPV infection played an important role in the process of cervical cancer progression, which was one of the important drivers of cervical cancer. The HPV gene integrated into the cervical cell genome, then changed the cervical cell genome state, eventually creating important changes in the biological behavior of cervical cells, such as changing the normal immune state of cervical cells, leading to the immune escape of cervical pathological cells. Many studies have confirmed that PD-L1 on the surface of cervical cancer cells was an important molecular marker of tumor immunity. Therefore, the relationship between HPV gene integration into the cervical cell genome and PD-L1 expression of cervical cells was explored, which could help further clarify the role of HPV in the evolution of cervical lesions and provide new ideas for the prevention and treatment of cervical cancer.

## 2. Materials and Methods

### 2.1. Sample Source

The 40 cases frozen cervical cancer pathological specimens were collected from the specimen repository of the cancer prevention research institute in the Tumor Hospital Affiliated to Xinjiang Medical University. All cases were Chinese Uygur cervical cancer, all of have existing HPV infection which were proved by flow-through hybridization and gene chip (FHGC). The ethics committee of the Tumor Hospital Affiliated to Xinjiang Medical University approved the study and the consent procedure.

### 2.2. Experimental Procedure

#### 2.2.1. The Test of HPV Gene Integration

The basic technique of the study was performed with an Agilent SureSelect™ Target Enrichment System and the second-generation sequencing technology (the HiSeq 2000 sequencer of Illumina).

*(1) Chip Design and Order*. We designed the sequence-capture probes according to 20 types of HPV genome (6, 11, 16, 18, 31, 33, 35, 39, 42, 43, 44, 45, 51, 52, 53, 56, 58, 59, 66, and 68) sequences, and these probes were produced by Agilent Inc., USA. The enrichment chip was also ordered from Agilent Inc., USA.

*(2) DNA Extraction*. The DNA of 40 cases were extracted by a Genomic DNA Extraction Kit. Agarose gel electrophoresis and Qubit fluorometer were used to evaluate the DNA quality.

*(3) Sample Test*. DNA fragments 150–200 bp were obtained after ultrasonic fragmentation; then, these fragments were purified, end blunted, “A” tailed, and adaptor ligated. The products of ligation were amplified by PCR and constructed into a DNA sequencing library. Bioanalyzer 2100 (Agilent Technologies, USA) was used to quantify the concentration of the DNA library. Next, hybridization was performed according to the instruction of Target Enrichment Protocol. This process was carried out at 65°C for 24 h, and the untargeted fragments were then removed by washing buffer. These eluted fragments were amplified by PCR and were further processed to paired-end 100 bp read-length for sequencing by the HiSeq 2000 sequencer (Illumina Inc., USA).

*(4) Data Analysis*. First, clean reads were obtained through removing low-quality and duplicated reads, as well as adaptor-contaminated reads. Burrows-Wheeler Aligner (BWA) was used to align clean reads onto human (NCBI build 37, HG19) and HPV genome. The paired-end reads that can be mapped to human or HPV reference genome with both ends were removed. The remaining reads were paired-end assembled to reconstruct fragment sequences, which was used to locate the position of breakpoints more precisely. Subsequently, reference mapping was performed again to remap the paired-end assembled reads onto human and HPV genome using BWA. The position of a breakpoint was defined as the junction of human and HPV sequence in a paired-end assembled read. To minimize the impact of different sequencing data amount of each sample, the normalized support-reads number (Norm value) was introduced, which was equal to the supported reads number of each HPV breakpoint per million clean read pairs. The breakpoints with Norm value ≥ 2 were retained. The Sanger sequencing validation rate for the selected breakpoints was 82.5%. ANNOVAR was used to do the annotation for the integrated breakpoints.

#### 2.2.2. Immunohistochemistry of PD-L1

Monoclonal antibodies of PD-L1 were bought from Golden Bridge Biotechnology Co. Ltd (Beijing, China). H&E staining was used to assess the pathological slices. The detection method used was IHC streptavidin peroxidase and diaminobenzidine chromogenic detection. All slices were produced using the following procedures: washing in xylene, ethanol, steamed classification, boiling water to wash, dissolving endogenous peroxide physical activity by incubation with 3% H_2_O_2_, antigen repair, and dyeing. Phosphate-buffered saline (PBS), rather than the first antibody, was the negative control; the previous positive specimen was the positive control. For PD-L1 expression in cancer cells, we chose the nests of cervical cancer including more than 100 cancer cells and then calculated the proportion of positive cells (10% or less was negative, more than 10% was positive); the positive criterion is in the cytomembrane and cytoplasm or both at the same time appeared tan particles.

### 2.3. Statistical Analysis

The Genesis analysis tool of webgenstal (http://www.webgestalt.org) and DAVID (https://david-d.ncifcrf.gov/) were used to complete the Gene Ontology categories and Kyoto Encyclopedia of Genes and Genomes (KEGG) gene function pathway categories. Multivariate logistic regression analysis was used to evaluate the relationship between the number of gene integration, the integration in 9p24.1, and PD-L1 expression by SPSS 17.0 statistical software.

## 3. Result

### 3.1. The HPV Genome Detection of 40 Cases

The most frequent was HPV16 type, followed with HPV18 type, HPV33 type, HPV39 type, and HPV45 type; their detection rate was 87.5%, 45.0%, 32.5%, 32.5%, and 25.0%, respectively. The detailed HPV infection results were shown in Table 1. From Table 1, the multiple HPV infection rate was 92.5%, which was much higher than the FHGC test in clinical laboratory (35.0%). The other high-risk HPV types besides HPV16 and HPV18 also had higher detection rate than FHCG test.

### 3.2. HPV Integration in Different Human Genome Chromosomes

**Figure 1:**
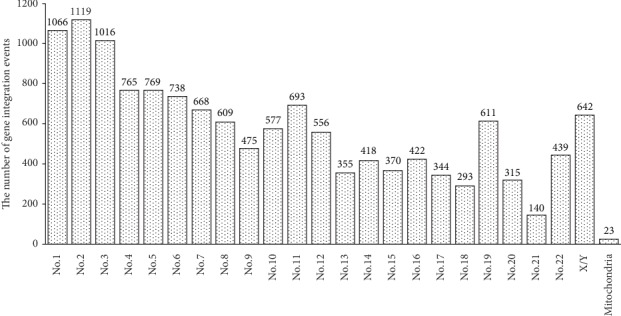
The number of HPV gene integration event in different human chromosomes.

13423 gene integration events were detected in 40 cases for 20 HPV gene subtypes, which involved 6867 human genes. The HPV gene was integrated into 24 human chromosomes and the mitochondrial genome; the detailed distribution numbers of integration events were in Figure 1. The most integrated events were detected in chromosome 2, which had 1119 integration events and had 8.34% of the total examined rate. The lowest number was found in chromosome 21, only 140 integration events.

**Figure 2:**
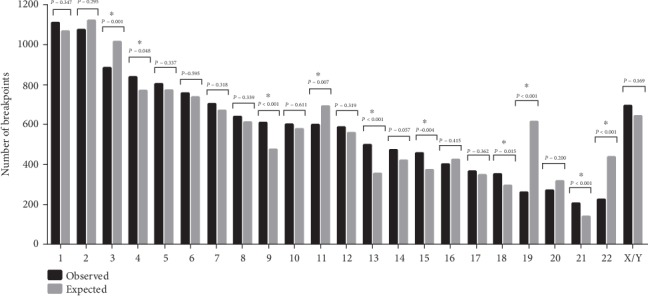
Comparison of chromosome integration distribution between observed and expected values.

Taking the length of every human chromosome as reference, the expected value of integration events that should exist on every chromosome was obtained, then compared to the number of integration events observed on each chromosome. The results are shown in Figure 2. In Figure 2, besides the mitochondrial chromosomes, the number between observation and expectation shows obvious difference in chromosomes 3, 4, 9, 11, 13, 15, 18, 19, 21, and 22, especially in chromosome 19, which had 611 observation integrated events, it is 2.34 times than 261 expectation events.

### 3.3. The Hot Integration Sites of HPV Integration in Human Genome

**Figure 3:**
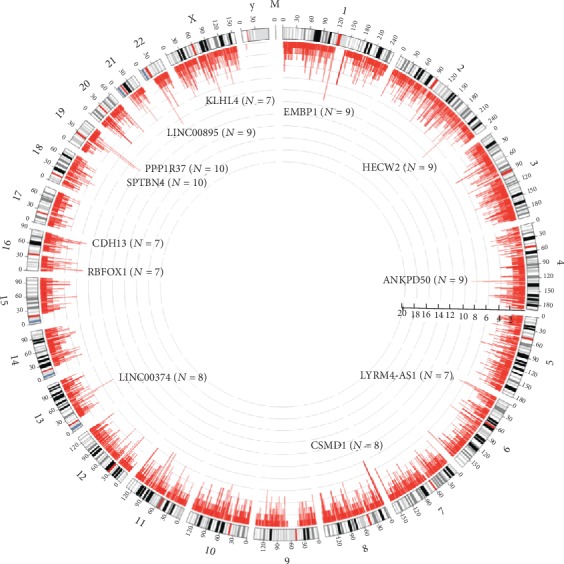
Distribution of breakpoints in the human genomes in 40 samples. Note: in the outer circle, each bar denotes the location of HPV integration into the 24 human chromosomes and the mitochondrial genome. Histogram axis units represent the number of samples, and outer DNA numbering is given in millions of bases. In the inner circle, each red bar depicts the frequency of HPV integration.

A total of 13423 integration events were detected in 40 samples, involving a total of 6867 human genes. Among these genes, the vast majority of genes could only be detected in one sample, and only a few genes could be detected in more than two samples. The distribution of every gene in human chromosome and the distribution of sample number obtained by detection were shown in Figure 3.

According to the results of statistical analysis, the top 10 genes in the occurrence frequency of 40 samples were PPP1R37, EMBP1, HECW2, ANKRD50, SPTBN4, LINC00895, CSMD1, LINC00374 and CDH13, RBFOX1, LYRM4-AS1, and KLHL4 genes. The detection rates were 25% (10/40), 22.5% (9/40), 22.5% (9/40), 22.5% (9/40), 22.5% (9/40), 22.5% (9/40), 20% (8/40), 20% (8/40), 17.5% (7/40), 17.5% (7/40), 17.5% (7/40), and 17.5% (7/40). In this study, these top 10 genes were referred to as hot integration gene sites of HPV gene integration.

### 3.4. Distribution of Integration Breakpoints in the HPV16 Genome

**Figure 4:**
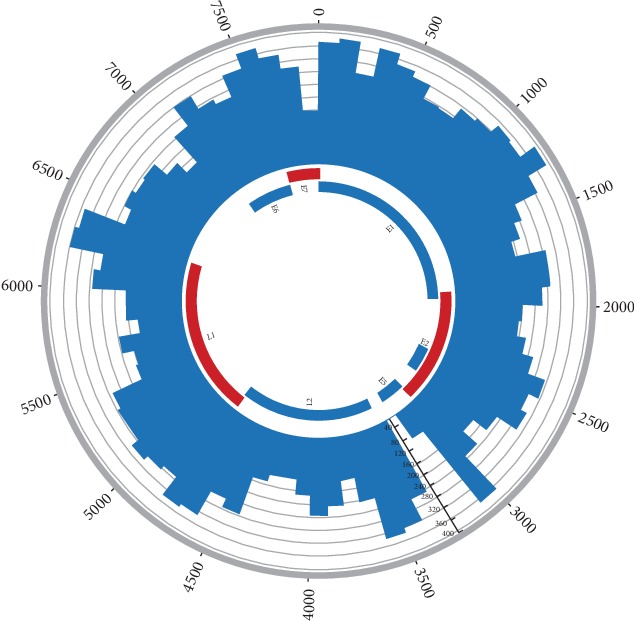
Distribution of breakpoints in the HPV16 genomes in 35 samples. Note: histograms (black) of the frequency of breakpoints in the samples were constructed for 100 bp intervals. Histogram axis units represent the number of breakpoints, and outer DNA numbering is given in bases. HPV genes with different functions are colored.

Among the 13423 integration events in the 40 samples, 5057 events were found to be caused by the integration of HPV16 gene into the host cell genome, which is from 35 samples. According to these 5057 integration events, the gene breakpoint distribution of HPV16 genome was analyzed, which is shown in Figure 4.

In Figure 4, every location of the HPV16 genome can be integrated into the host cell genome. According to the length of each HPV16 functional gene area, there were 81 gene breakpoints in every 100 bp intervals of E6 gene, which had the most frequent gene breakpoints. But there were 46 gene breakpoints in every 100 bp intervals of E5 gene, which was the least.

**Figure 5:**
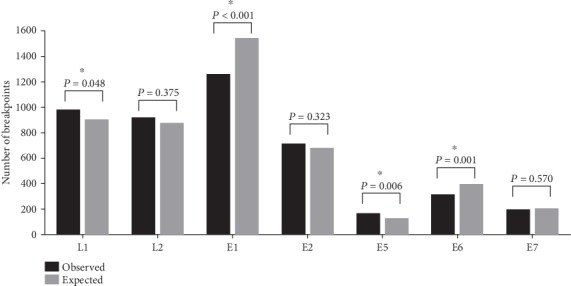
Comparison of the observed and expected numbers of breakpoints in HPV16 genome.

Taking the length of every functional gene of HPV16 genome as reference, the expected value of gene breakpoints that should exist in every functional gene region was obtained, then compared to the actual number of observed gene breakpoints. The results were shown in Figure 5. There was a statistical difference between the expected value and the observed value of the gene breakpoints in L1, E1, E5, and E6 genes. The observed gene breakpoints of E1 and E6 genes were much higher than the expected value, which were 1.23 times and 1.26 times, respectively. However, the observed number of gene breakpoints in E5 gene was much lower than the expected value, accounting for only 72.2% of the expected value.

### 3.5. Functional and Pathway Enrichment Analysis of the Integration Sites

**Figure 6:**
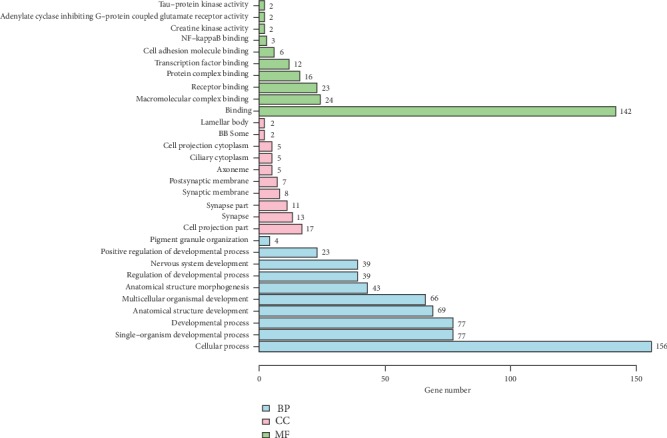
GO categories assigned to all 6867 integration genes. Note: the *y*-axis represents the count of genes. The genes were categorized according to the annotation of GO, and the number of each category is displayed based on biological process (BP), cellular components (CC), and molecular functions (MF).

The results of Gene Ontology categories are showed in Figure 6 and Table 2; GO categories showed that binding of the molecular function was the main part in the functional level of 6867 genes, which included 142 genes. Meanwhile, cellular process of the biological process was the key component, which had 156 genes. But for the cellular component categories, there was no preponderant component.

**Figure 7:**
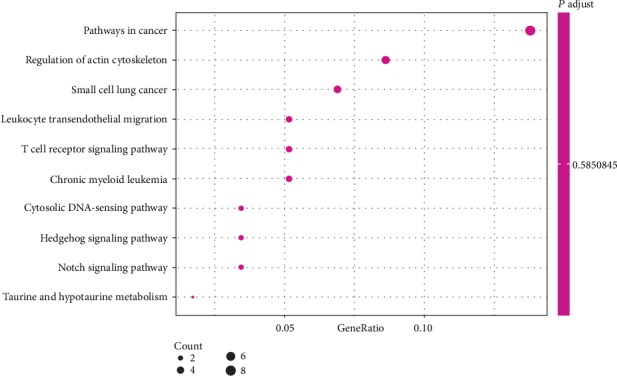
The dotplot picture of the top 10 enrichment KEGG categories. Note: each point in the figure represents a KEGG pathway, whose name is shown in the left coordinate axis. The abscissa is the GeneRatio, indicating the ratio of the genes annotated into the pathway in 58 genes and that of the species genes annotated into the pathway.

Kyoto Encyclopedia of Genes and Genomes (KEGG) pathway enrichment analysis was performed on 58 human genes, whose integration sites were detected more than 20 times. The top 10 enrichment pathways of gene function pathway categories are showed in Figure 7 and Table 3. The pathways in cancer was the top enrichment pathway, which included 8 genes, including RELA, BCL2, LAMA5, CBLB, FGF18, HDAC2, RXRA, and FGF14, which accounted for 14% of gene number in the pathways.

### 3.6. Multifactor Analysis between the Number of Gene Integration, the Integration in 9p24.1, and PD-L1 Expression

**Figure 8:**
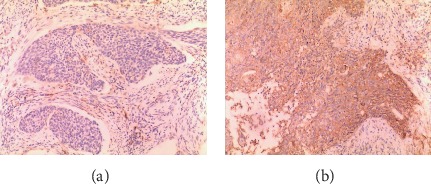
Expression of PD-L1 in cervical cancer cell. Notes: (a) negative and (b) positive expression of PD-L1 in cervical cancer cells (×100 amplification).

The positive and negative PD-L1 expression in cervical cancer cells was present in 25 cases (62.5%) and 15 cases (37.5%), respectively (example in Figure 8). The number of gene integration and the gene integration status in 9p24.1 were shown in Table 4. The corresponding logistic regression expression was

(1) $$\begin{array}{c} \text{Logit P}=-2.188+0.012\times \text{Number of gene integration}+2.815\times \text{Gene integration in }9\text{p}24.1. \end{array}$$

Table 5 shows the results of multivariate logistic regression analysis. Therefore, the number of gene integration and the gene integration in 9p24.1 were the risk factors which were significantly associated with increased risk of PD-L1 expression.

## 4. Discussion

The analysis methods of the Agilent SureSelect™ Target Enrichment System and the second-generation sequencing technology (the HiSeq 2000 sequencer of Illumina) included to one of the high-throughput viral integration detection (HIVID) had proved to be very useful test methods [14, 15]. In the study, the Agilent SureSelect™ Target Enrichment Chip was designed by Agilent company, which also had its patent right, which ensured the accuracy of test results. The chip included 20 HPV types; it had firstly reported so many HPV type integration detection. Because in the previous study, most HPV type integration detection only included 11 HPV types [16], the study greatly increased the detection range of HPV types.

At present, the current clinical detection methods of HPV type were FHGC, real-time fluorescence quantification, and so on, which were the principle of various commercial HPV type detection kits. For 40 specimens, the retrospective results of HPV type detection were given by commercial HPV type detection kits, which was approved by CFDA, and the detection method was FHGC. In the retrospective results, the HPV subtypes were mainly HPV16 and HPV18; the HPV multiple infection ratio was 35% (14/40), which was exactly consistent with the previous reports [17]. But by the HIVID method, for 40 specimens, HPV16 was the most frequently detected, followed by HPV33, HPV39, HPV18, and HPV45. 37 samples were HPV multiple infection, which accounted for 92.5%. So the results between the new HIVID method and FHGC were very different. The new HIVID method depended on enrichment chip and second-generation sequencing technology, which could improve the sensitivity of detection. Because the high HPV multiple infection ratio was found by HIVID, it may be possible to make a large correction for the existing opinion about HPV typing detection; we must pay more attention to the HPV multiple infection, the infection pattern may exist far beyond our imagination.

By analyzing the specific integration sites of HPV genes, it was found that the integration sites involved all chromosomes of human genome, with a total of 6867 human genes, indicating the randomness of HPV gene integration. PPP1R37, EMBP1, HECW2, ANKRD50, SPTBN4, LINC00895, CSMD1, LINC00374, CDH13, RBFOX1, LYRM4-AS1, and KLHL4 were the high-frequency integration sites; compared to the hotspots reported by Zheng et al. [16], there were significant differences in the hotspots. In their report, POU5F1B, FHIT, KLF12, HMGA2, KLF5, LRP1B, LEPREL1, DLG2, and SEMA3D were the hotspots. But the high-frequency integration sites reported by Li et al. were FHIT, CASC8, KLF5, LINC00392, RAD51B, CASC21, ERBB2, TP63, TEX41, RAP2B, and MYC [17]. These high-frequency integration sites were quite different, which indicates that there were different high-frequency integration sites in different sample sources. With the development of detection technology, new high-frequency integration sites are constantly found. Existing studies have reported that some Common Fragile Sites (CFS) widely exist in human chromosomes [18], which are often the common integration sites of various carcinogenic virus genes into the human genome including HPV. The reported CFS were 40, and two high-frequency integration sites in the study belong to CFS, such as CSMD1 and CDH13. The result verified the assertion that the CFS also were the hotspots of the integration of HPV gene.

In Figure 4, it was found that breakpoints could occur in any part of the viral genome, perhaps enabling the virus to adapt to the changing environment during carcinogenesis. The E1 gene functions had most breakpoint number in every 100 bp intervals, and E2 gene functions had the least breakpoint number in every 100 bp intervals. In the previous report, Zheng et al. also showed the same phenomenon [16]. First, E1 involves viral DNA replication, which plays a key role when the virus begins to replicate. Because E1 had the most breakpoint number, the virus must be different to replicate [19–22]. Then, L1 gene also had some breakpoints, and the detection target for most commercial HPV type detection kits was L1 gene. So the two reasons could lead to missed detection due to HPV genomic integration [23, 24].

By the functional and pathway enrichment analysis, it could be found that the HPV gene integration could mainly affect the physiological process of host cells and could also affect pathways in cancer. The GO and KEGG categories analyzed the integration gene sites, which was the first comprehensive analysis about the integration landscape. The pathways in cancer were a cell signal transduction system which played an important role in cell carcinogenesis and cancer evolution. Because HPV gene integration had the deepest level of impact about the pathways in cancer, so it could be supposed that the HPV gene integration into the cervical cells could cause the occurrence and progression of cervical cancer.

As for the relationship between HPV infection and PD-L1 expression in cervical cells, it is still a great controversy. Most reports showed that HPV infection promoted the high expression of PD-L1 [25–27]. Previous studies of our research group have also confirmed that high-risk HPV infection had higher levels of PD-L1 expression in cervical cancer patients [28]. In 2016, Keisuke et al. demonstrated that gene rearrangement on the 3′-UTR of CD274 gene in tumor cells because of HPV gene integration would lead to abnormal expression of PD-L1 [29]. Also, some reports explored that the change of 9p24.1 could increase the high expression of PD-L1, because of 9q24.1 including many genes such as JAK2 and PDCD1LG2, which were closely related to PD-L1 expression [30]. Meanwhile, the latest research reported that the tumor-specific superenhancer in 9q24.1 could increase the expression of PD-L1 and PD-L2 [31]. So these genes in 9q24.1 were changed by HPV-DNA integration, which may create some kind of effect to affect PD-L1 expression. PD-L1 antibody drugs are considered as the star of tumor immunotherapy, and it has been proved that tumor mutation burden (TMB) was closely related to the efficacy of PD-L1 antibody drugs in a variety of tumor [32, 33]. The integration of viral genes into tumor cells has an effect on the genome of tumor cells, which was also a mutation burden in theory, so we called it viral gene integration burden (VIB). Existing theory proved that the potential cause of TMB to improve the efficacy of tumor immunotherapy is the production of neoantigen. But by the study, we found that the VIB similar to TMB in cervical cancer was closely related to PD-L1 expression. The number of gene integration and the presence of gene integration in 9p24.1 were both risk factors that promote the high expression of PD-L1. It was proved that this phenomenon of VIB not only promoted the production of neoantigen but also improved the expression of PD-L1 in tumor cells, thereby improving the efficacy of PD-L1 antibody immunotherapy.

## 5. Conclusion

The chip enrichment combined with the second-generation sequencing technology could significantly improve the detection sensitivity of HPV integration sites; multiple infectious problem was worthy of in-depth study, the HPV gene integrated into the human genome was universal and random, but also, there were some hotspots in human genome, and the hotspots in Chinese Uygur cervical cancer women were different from the other Chinese population. The breakpoints of HPV genome could lead to missed detection of the commercial HPV type detection kits. The integration of HPV genes into the human genome could affect pathways in cancer, which was a key step in the development of cervical cancer. The gene integration especially the integration existing on 9p24.1 could affect the expression level of PD-L1. The VIB should regulate the high expression of PD-L1 in some cancer cells because of oncogenic virus such as HBV and EB.

## Figures and Tables

**Table 1:** Comparison of HPV type infection results by high-throughput viral integration detection (HIVID) and flow-through hybridization and gene chip (FHGC).

Sample no.	HIVID	FHGC	Sample no.	HIVID	FHGC
1	16, 33, 39	16	21	18	18
2	16, 33	16	22	18, 45	18
3	16, 33, 39	16, 33	23	16, 56	16
4	16, 33, 39, 45	16, 45	24	18, 52	18
5	16, 33	16	25	16, 18, 58	16, 18
6	16, 33, 39	16	26	16, 33, 52	16
7	16, 18, 33, 39	16, 18	27	18, 45	18
8	16, 33, 39	16	28	16, 39	16
9	16, 33, 45	16	29	16, 18, 33	16
10	16, 33	16	30	16, 45	16
11	16, 18, 45	16, 18	31	16, 18	16, 18
12	16, 18, 39	16, 18	32	16, 18, 39	16, 18
13	16, 18	16, 18	33	16, 33, 39	16
14	16, 45	16	34	16, 45	16
15	16, 39	16	35	18, 33	18
16	16, 39, 45	16	36	16, 18	16, 18
17	16, 39	16	37	16, 18, 33	16, 18
18	16, 18	16, 18	38	16, 39	16
19	16	16	39	16, 18, 45	16, 18
20	16	16	40	16, 18	16, 18

**Table 2:** The GO analysis of breakpoints.

ID	Description	*P* value	*P* adjust
GO:0005488	Binding	0.000282293	0.035209594
GO:0044877	Macromolecular complex binding	0.000659256	0.101525445
GO:0005102	Receptor binding	0.001913446	0.124504264
GO:0032403	Protein complex binding	0.004070801	0.197687118
GO:0008134	Transcription factor binding	0.001408885	0.124504264
GO:0050839	Cell adhesion molecule binding	0.004394358	0.197687118
GO:0051059	NF-kappaB binding	0.002155918	0.124504264
GO:0004111	Creatine kinase activity	0.001161827	0.124504264
GO:0001640	Adenylate cyclase-inhibiting G protein-coupled glutamate receptor activity	0.001617015	0.124504264
GO:0050321	Tau-protein kinase activity	0.004935012	0.197687118
GO:0044463	Cell projection part	0.001623765	0.132806474
GO:0045202	Synapse	0.004266516	0.144634899
GO:0044456	Synapse part	0.003134076	0.132806474
GO:0097060	Synaptic membrane	0.002565895	0.132806474
GO:0045211	Postsynaptic membrane	0.003117983	0.132806474
GO:0005930	Axoneme	0.000884334	0.099929701
GO:0097014	Ciliary cytoplasm	0.000884334	0.099929701
GO:0032838	Cell projection cytoplasm	0.002257255	0.132806474
GO:0034464	BBSome	0.003945579	0.144634899
GO:0042599	Lamellar body	0.005714065	0.176097096
GO:0009987	Cellular process	4.38*E*-05	0.016489135
GO:0044767	Single-organism developmental process	6.59*E*-06	0.006614675
GO:0032502	Developmental process	1.25*E*-05	0.007498198
GO:0048856	Anatomical structure development	3.32*E*-05	0.014273002
GO:0007275	Multicellular organismal development	5.04*E*-05	0.016552061
GO:0009653	Anatomical structure morphogenesis	2.96*E*-05	0.014273002
GO:0050793	Regulation of developmental process	4.64*E*-06	0.006614675
GO:0007399	Nervous system development	8.81*E*-06	0.006626031
GO:0051094	Positive regulation of developmental process	5.50*E*-05	0.016552061
GO:0048753	Pigment granule organization	6.81*E*-05	0.018642610

**Table 3:** The KEGG analysis of breakpoints.

ID	Description	*P* value	*P* adjust^∗^
hsa05200	Pathways in cancer	0.009462254	0.585084485
hsa05222	Small cell lung cancer	0.013923376	0.585084485
hsa05220	Chronic myeloid leukemia	0.034615567	0.585084485
hsa04810	Regulation of actin cytoskeleton	0.058285601	0.585084485
hsa04330	Notch signaling pathway	0.077633762	0.585084485
hsa04660	T cell receptor signaling pathway	0.089578675	0.585084485
hsa00430	Taurine and hypotaurine metabolism	0.094228757	0.585084485
hsa04340	Hedgehog signaling pathway	0.104655279	0.585084485
hsa04623	Cytosolic DNA-sensing pathway	0.104655279	0.585084485
hsa04670	Leukocyte transendothelial migration	0.107434188	0.585084485

^∗^All of FDR (adjusted *P* value) > 0.05, the potential reason was that the number of gene was too few, so the *P* value < 0.05 instead of FDR < 0.05 was the appropriate statistical analysis criteria to analyze the final result.

**Table 4:** The PD-L1 expression, gene integration number, and the gene integration in 9p24.1 of 40 cases.

No.	PD-L1 expression	Gene integration number	Gene integration in 9p24.1
1	Negative	29	No
2	Negative	38	Yes
3	Positive	99	No
4	Positive	445	Yes
5	Positive	347	Yes
6	Positive	382	No
7	Negative	56	No
8	Positive	110	No
9	Negative	302	No
10	Positive	152	No
11	Negative	204	No
12	Positive	102	Yes
13	Positive	309	No
14	Positive	285	Yes
15	Positive	445	Yes
16	Positive	561	Yes
17	Positive	134	No
18	Negative	159	No
19	Positive	168	Yes
20	Positive	179	Yes
21	Negative	47	No
22	Negative	52	No
23	Positive	574	No
24	Negative	74	No
25	Positive	2927	Yes
26	Positive	129	Yes
27	Positive	283	No
28	Negative	104	No
29	Positive	107	No
30	Negative	109	No
31	Positive	127	Yes
32	Negative	118	No
33	Positive	123	No
34	Positive	109	Yes
35	Positive	179	Yes
36	Positive	217	Yes
37	Negative	220	No
38	Negative	101	No
39	Positive	3412	Yes
40	Negative	72	No

**Table 5:** Results of multivariate logistic regression analysis.

Risk factors	*β*	Wald*χ*^2^ value	*P* value	OR value	OR (95% CI)
Gene integration number	0.012	4.693	0.030	1.012	1.001-1.023
Gene integration in 9p24.1^∗^	2.815	5.773	0.016	17.317	1.691-177.213
Constant	-2.188	5.613	0.018	0.012	

CI: confidence interval; OR: odds ratio. ^∗^Compared to no gene integration in 9p24.1.

## Data Availability

The data used to support the findings of this study are available from the corresponding author upon request.
